# The Influence of Political Trust on Attitude towards Public Participation in China

**DOI:** 10.1371/journal.pone.0318221

**Published:** 2025-03-10

**Authors:** Manfei Cui, Yong Li

**Affiliations:** 1 College of Economics & Management, Zhejiang University of Water Resources and Electric Power, Hangzhou, China; 2 School of Marxism, Shanghai Maritime University, Shanghai, China; University of Exeter, UNITED KINGDOM OF GREAT BRITAIN AND NORTHERN IRELAND

## Abstract

This study aims to examine the impact of political trust on non-institutionalized public participation in China. Using comprehensive survey data from the Chinese General Social Survey (CGSS), this study presents two key research findings. First, this study finds a non-linear relationship between political trust and attitude towards non-institutionalized public participation. Generally, political trust has a negative effect on attitude towards non-institutionalized public participation. However, political trust exerts a positive impact on attitude towards non-institutionalized public participation under notably high political trust circumstances. Second, this study identifies the moderating role of social injustice experience in the relationship between political trust and the attitude towards non-institutionalized public participation. When high expectations associated with high trust are broken by a social injustice experience, this high political trust circumstance is more likely to result in dissatisfaction and positive attitude towards non-institutionalized public participation. This study contributes to deepening the understanding of the pattern of how political trust influences non-institutionalized public participation attitude in China. Valuable implications are also presented.

## Introduction

Political trust is a crucial indicator of regime support [1]. In China, robust economic performance and a sequence of reforms have promoted people’s trust in government [2,3]. Previous research shows that, compared with other countries, China has maintained a high level of political trust for decades [3–5]. Prior studies also indicate the expansion of public participation in China [6,7]. It is widely agreed that distrust is correlated with non-institutionalized public participation [7,8]. However, Li and O’Brien [9] put forward the explanation of hierarchical government trust which refers to individuals who believe the central government rather than the local government would participate in non-institutionalized public participation. Hierarchical government trust is a typical characteristic of Chinese people’s political trust [3,10]. It is reported that citizens believe that when they face problems with local government they can appeal to higher levels of government for help [11]. This hierarchical government trust theory explains the phenomenon whereby some non-institutionalized public participants with high political trust. Based on the above discussion, there is an absence of research regarding whether there is a non-linear relationship between political trust and the non-institutionalized public participation.

Utilizing data from CGSS 2006, this study aims to analyze the influence of political trust on non-institutionalized public participation and contribute to improving social governance. First, this study reveals that political trust has both positive and negative impacts on attitude towards non-institutionalized public participation. Second, this study discovers the moderating role of social injustice experience in the relationship between political trust and attitude towards non-institutionalized public participation. When the high expectations brought by high trust are broken by a social injustice experience, this psychological setback could result in dissatisfaction and positive non-institutionalized public participation attitude.

### Political trust and attitude towards non-institutionalized public participation

Non-institutionalized public participation refers to individuals opting to express their viewpoints or resistance bypassing the legitimate institutional channels [9,12]. In this study, we used attitude towards non-institutionalized public participation as a proxy variable for non-institutionalized public participation. Attitude refers to an individual’s stable psychological tendency, and contains their action orientation [13,14]. Generally speaking, non-institutionalized public participation participants are difficult to observe directly [4]. Even if it is possible to observe the behavior of non-institutionalized public participants, it is difficult to find a comparison group, leading to the problem of sample selection bias. By using the concept of attitude towards non-institutionalized public participation, the relationship between political trust and non-institutionalized public participation can be investigated more comprehensively and with less bias.

Political trust is defined as the citizens’ belief that the government will work to produce outcomes that are consistent with their expectations [15–18]. People who trust the government are more likely to comply with the government policies [19]. In contrast, a low level of political trust may lead to citizens’ dissatisfaction with the government [20]. Distrust in a government has been found to be associated with the approval of non-institutionalized public participation [21,22].

People’s trust in government depends on both perceptions and expectations [23]. According to Bhattacharya et al. [24], trust is generally regarded as a kind of dependency relationship, indicating a positive expectation. In this regard, high political trust usually co-exists with high expectations. While the capacity of the government is not infinite, and the governments’ response sometimes may lag behind. This gap between these two may give rise to dissatisfaction and lead to non-institutionalized public participation in urban China [25]. As Yang points out, a high level of trust can be both a blessing and a curse, because it is difficult to meet high expectations consistently [26].

In addition, political efficacy theory provides the explanation of rational choice mechanism between political trust and non-institutionalized public participation. Chen [27] argues that some poor people have little bargaining power unless they are able to engage in non-institutionalized public participation effectively. In other words, non-institutionalized public participation is a tactic based on people’s rational choices. Many studies suggest that some people believe that the central government sides with them in regulating unlawful local cadre behavior [28–30]. Li [8] believes that citizens with high political trust tend to engage in non-institutionalized public participation to safeguard their own interests and change the status quo. Based on the political efficacy theory, Hu [31] finds that individuals with high political efficacy may rationally choose non-institutionalized participation when they perceive institutional channels as inadequate for addressing their concerns. In this view, the relationship between political trust and attitude towards non-institutionalized public participation may no longer be negatively linear shaped.

Based on the above discussion, this study suggests that there is a threshold in the relationship between political trust and non-institutionalized public participation attitude. It is generally agreed that political trust tends to reduce non-institutionalized public participation attitude. However, political trust can also have a positive influence on attitude towards non-institutionalized public participation under high expectation mechanism and political efficacy mechanism. Hence, Hypothesis 1 is formulated as follows:

Hypothesis 1 (H1)The relationship between political trust and attitude towards non-institutionalized public participation is non-linear.

### The role of social injustice experience

According to the previous study, social injustice is a crucial antecedent of non-institutionalized public participation [32]. Social injustice refers to the evaluation of a situation as unjust or inequitable, and the evaluation is not merely cognitive but emotional. From the psychological perspective, social injustice is viewed as a “stimulus” [33]. Previous studies have identified the moderating role of social injustice experience [34–38]. Thus, in this study, we postulate that social injustice experience exerts a moderating effect on the relationship between political trust and non-institutionalized public participation attitude. For citizens who have suffered from social injustice, in the first case for the low political trust group, their low political trust will breed dissatisfaction when confront with injustice. Injustice experience together with low political trust mutually strengthen the citizens’ positive attitude towards non-institutionalized public participation. For citizens who have high political trust and have suffered social injustice, their social injustice experience is the thorn that punctures their high expectations. The disparity between high expectations and the injustice will cause psychological setback and dissatisfaction and provides an incentive to non-institutionalized public participation [8,9]. In light of the above reasoning, the following hypothesis is developed:

Hypothesis 2 (H2)Social injustice experience plays a moderating role in the relationship between political trust and attitude towards non-institutionalized public participation.

## Methods

This study uses data from the China’s General Social Survey (CGSS) 2006, which is a national, large-scale household survey conducted by the Renmin University of China and Hong Kong University of Science and Technology. CGSS is an ongoing academic survey project initiated in 2003. Using a multi-stage stratified random sampling method, CGSS aims to gather longitudinal data on various aspects of Chinese society, providing essential information for social science research. CGSS 2006 wave is the only one with all the variables investigated in this study. Although the data are from 2006 the research conclusions of these data still have certain value for us to explore the impact of political trust on non-institutionalized public participation. In this study, urban sample data are selected and the number of valid samples is 5687. The reasons why we select urban sample are as follows. First, political trust is measured by the questions related to urban life in this study. In the dataset of CGSS 2006, urban and rural are collected separately. Second, previous study suggests that there are significant differences between urban and rural areas in terms of political trust and political participation [3]. We aim to engage in dialogue with some literature on urban participation research [39–41].

The dependent variable, i.e., attitude towards non-institutionalized public participation, is measured by asking “Do you support using the non-institutionalized approaches, such as strikes, to express your views on your own interests?” The options are on the Likert 5-point scale, ranging from 1 = “strongly disagree” to 5 = “strongly agree”, with a Cronbach’s α of 0.807.

Political trust is measured by asking “In the following topics, when the various channels of information are inconsistent, how much do you trust the government?” The topics include housing prices, stock market trends, domestic corruption, the gap between rich and poor, the employment rate among college students, public security, and mining accidents. The options are on the Likert 5-point scale, ranging from 1 = “not trust at all” to 5 = “a great deal of trust”, with a Cronbach’s α of 0.883.

Social injustice experience is a dummy variable, measured by asking “In the past five years, have you been unequally treated in the following issues, for instance, property disputes, and unemployment subsidies?” Respondents choosing “yes” are coded as 1, and otherwise as 0.

Some demographic factors are added in the investigation. Marien et al. [42] find that younger people are more positive in public participation. According to Coffé and Bolzendahl [43], men are more likely to engage in non-institutionalized public participation. Previous study shows that CPC membership is related to public participation [44]. As suggested in existing research, education level is a determinant of public participation [42]. Prior research indicates that employment status is an influential factor of public participation [45]. Zheng et al. [46] and Shi et al. [47]suggest that life satisfaction is associated with public participation. Thus, these variables are selected as the control variables. (1) age, ranging from 18 to 70 years old; (2) gender; (3) CPC (the Communist Party of China) membership; (4) education in years; (5) employment status; (6) life dissatisfaction, ranging from 5 (very dissatisfied) to 1 (very satisfied). The descriptive statistics for all the variables are presented in Table 1.

**Table 1:** Descriptive statistics and the pearson correlation.

	Mean	SD	1	2	3	4	5	6	7	8	9
1. Age	42.390	13.449	1.00								
2. Gender	0.462	0.499	-0.009	1.00							
3. CPC	0.09	0.282	0.189**	0.173**	1.00						
4. Education	9.080	3.631	-0.389**	0.079**	0.151**	1.00					
5. Employment status	0.650	0.478	-0.361**	0.207**	-0.003	0.222**	1.00				
6. Life dissatisfaction	2.689	0.611	0.139**	-0.074**	-0.071**	-0.190**	-0.246**	1.00			
7. Political trust	3.734	0.805	0.011	-0.031*	-0.006	-0.021	-0.003	-0.048**	1.00		
8. Social injustice experience	0.120	0.321	0.070**	0.027*	0.023	-0.065**	-0.029**	0.158**	-0.057**	1.00	
9. Attitude towards non-institutionalized public participation	2.541	1.051	-0.049**	0.028*	-0.052**	0.006	-0.002	0.117**	-0.126**	0.064**	1.00

Notes:

* p <  0.05,

**p <  0.01; N =  5687

## Results

Table 2 presents the results of the five multiple linear regression models, in which the attitude towards non-institutionalized public participation is set as the dependent variable. Model 1 includes only the control variables. Subsequently, the political trust and political trust square are added in Models 2 and 3. Social injustice experience is added in Model 4. Model 5 is enlarged by the interaction item of political trust and social injustice experience based on Model 4.

**Table 2:** Predicting the attitude towards non-institutionalized public participation.

	Model 1	Model 2	Model 3	Model 4	Model 5
**Control variables**					
Age	-0.004*** (0.005)	-0.004*** (0.001)	-0.004*** (0.001)	-0.005*** (0.001)	-0.005*** (0.001)
Gender	0.096*** (0.029)	0.090** (0.029)	0.092*** (0.029)	0.045** (0.028)	0.089** (0.029)
CPC	-0.137** (0.048)	-0.134** (0.048)	-0.137** (0.048)	-0.113** (0.043)	-0.138** (0.048)
Education	0.004 (0.005)	0.002 (0.005)	0.003 (0.005)	-0.003 (0.005)	0.003 (0.005)
Employment status	-0.015 (0.031)	-0.020 (0.031)	-0.016 (0.031)	0.029 (0.037)	-0.017 (0.031)
Life dissatisfaction	0.312*** (0.033)	0.296*** (0.033)	0.297*** (0.033)	0.282*** (0.033)	0.282*** (0.033)
**Independent variables**					
Political trust Political trust square		-0.159*** (0.017)	-0.572*** (0.121) 0.058*** (0.017)	-0.564*** (0.120) 0.058*** (0.017)	-0.499*** (0.122) 0.052** (0.017)
Social injustice experience				0.134** (0.043)	0.114** (0.044)
**Interaction variable**					
Political trust × Social injustice experience					-0.146*** (0.033)
Intercept	1.937*** (0.119)	2.589*** (0.139)	3.272*** (0.241)	3.278*** (0.115)	3.120*** (0.245)
N	5687	5687	5687	5687	5687
Adjusted R^2^	0.021	0.035	0.037	0.039	0.041
∆R^2^	/	0.014	0.002	0.002	0.002
F value	21.416***	30.723***	28.434***	26.378***	25.057***

Notes: Entries are unstandardized OLS regression coefficients, with standard errors shown in parentheses beneath.

* p <  0.05,

**p <  0.01,

***p <  0.001.

As displayed in Table 2, Model 1 shows the influence of all of the control variables on attitude towards non-institutionalized public participation. Age has a significantly negative effect on attitude towards non-institutionalized public participation (M1, *β*= -0.004, p <  0.001), which may be due to the fact that older people behave more conservatively [3]. Gender has a significantly negative effect on attitude towards non-institutionalized public participation (M1, *β*= 0.096, p <  0.001). Namely, males have a more positive attitude towards non-institutionalized public participation than females, which is in line with the previous studies [7,43]. The members of the CPC have a more negative attitude towards non-institutionalized public participation (M1, *β*= -0.137, p < 0.01) compared with counterparts. Furthermore, dissatisfaction with life has a significantly positive effect on the attitude towards non-institutionalized public participation (M1, *β*= 0.312, p < 0.001). Citizens with dissatisfaction with life are more inclined to engage in non-institutionalized participation.

### Political trust has a non-linear effect on the attitude towards non-institutionalized public participation

**Fig 1:**
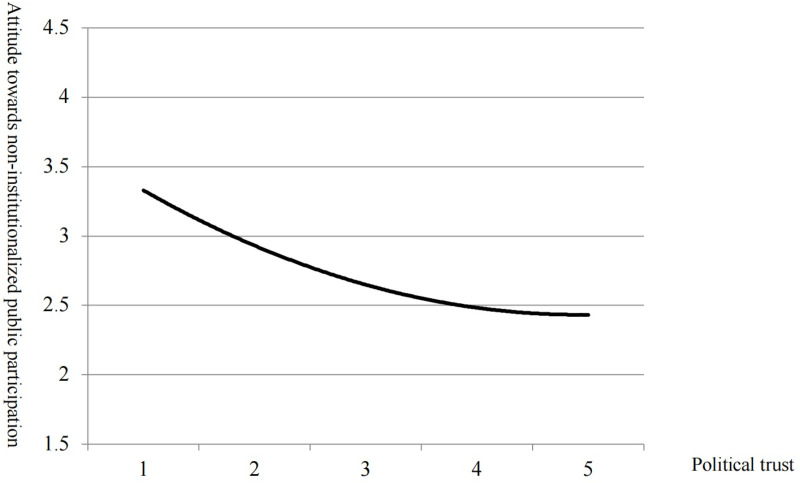
The relationship between political trust and attitude towards non-institutionalized public participation.

As shown in Model 3 in Table 2, the coefficients of political trust and political trust square are both significant (M3, β= -0.572, p <  0.001; M3, β =  0.058, p <  0.001), indicating that political trust has a significant non-linear effect on the attitude towards non-institutionalized public participation. Fig 1 demonstrates the fitting curve of political trust and attitude towards non-institutionalized public participation. Generally, the lower political trust, and more positive the attitude towards non-institutionalized public participation will be (Fig 1). However, when the level of political trust is notably high, the higher the political trust becomes, the more positive attitude towards non-institutionalized public participation will be. H1 is verified.

### The moderating role of social injustice experience

**Fig 2:**
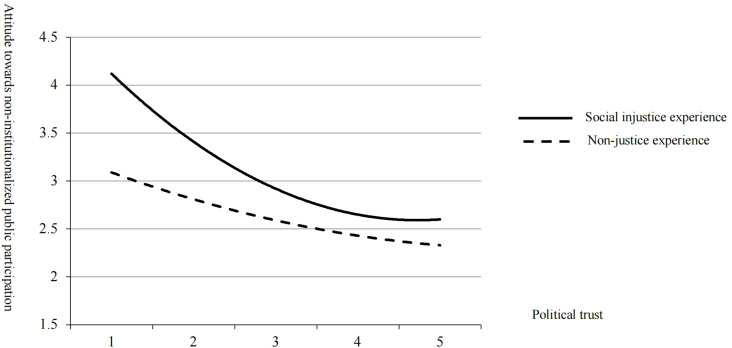
The moderating effect of social injustice experience in the relationship between political trust and attitude towards non-institutionalized public participation.

From Table 2, Model 4 shows that the experience of social injustice has a significantly positive effect on attitude towards non-institutionalized public participation (M4, β= 0.134, p < 0.01) after controlling for the other variables. Moreover, the interaction term of social injustice experience and political trust has a significantly effect on attitude towards non-institutionalized public participation (M5, *β=* -0.146, p < 0.001), indicating that social injustice experience plays a moderating role in the relationship between political trust and attitude towards non-institutionalized public participation. H2 is supported. Fig 2 illustrates the curvilinear relationships. The U-shaped covariant curve between political trust and attitude towards non-institutionalized public participation becomes steeper under the condition of social injustice experience (Fig 2).

## Discussions and conclusions

Through conducting analysis based on nationwide data from CGSS 2006, this study presents the following conclusions. First, this study finds that political trust has a non-linear effect on the attitude towards non-institutionalized public participation. Within a certain range of political trust, the more citizens trust the government, the more negative their attitude towards non-institutionalized public participation will be. Nevertheless, under a notably high level of political trust, a turning point is reached in the relationship between political trust and attitude towards non-institutionalized public participation, and the direction of the influence changes from negative to positive. Second, this study finds that social injustice experience positively influences attitude towards non-institutionalized public participation. Compared with non-injustice experience, citizens who have experienced injustice show a more positive attitude towards non-institutionalized public participation. In addition, this study reveals that social injustice experience plays a moderating role in the relationship between political trust and attitude towards non-institutionalized public participation.

Based on the research findings, the study presents several implications and suggestions as follows. First, this study deepens the understanding of the relationship between political trust and non-institutionalized public participation attitude and draws an integrated and comprehensive picture of the relationship between them in China. This study reveals the double-edged effect of political trust on attitude towards non-institutionalized public participation in China. As Nye et al. [23] point out, people’s trust in the government depends on both their perception and expectation. From the perspective of perception, political trust has an inhibiting effect on the attitude towards non-institutionalized public participation. Less political trust has been found to be associated with more positive attitude towards non-institutionalized public participation [21,22,29]. However, from the perspective of expectation, the high and unfulfilled expectation may lead to a positive non-institutionalized public participation attitude.

Second, this study suggests that social injustice experience is an influential factor of attitude towards non-institutionalized public participation, and also plays a moderating role in the relationship between political trust and non-institutionalized public participation attitude. Compared with citizens who have not been treated unjustly, those who have injustice experience display a more positive attitude towards non-institutionalized public participation. For those with high political trust, when their high expectations are not met, the psychological setback between their high expectations and their experience of injustice leads to a positive attitude towards non-institutionalized public participation.

Third, to promote institutionalized public participation, rational and adaptive expectations of the public should be guided by the governments. It is suggested that, instead of building a government that takes care of everything, shaping an image of a responsive government better aligns with the needs of modernization. Furthermore, the attitude towards non-institutionalized public participation is significantly driven by social injustice experience. Building a more just and equal society is of utmost importance. For instance, enforcing the rule of law, establishing effective channels for gathering public feedback, facilitating the communication between the government and the public, and promoting good governance are essential measures.

## Limitation

The limitations and future directions of this study can be summarized in two points. First, this study conducts a cross-sectional study to explore the relationship between political trust and public participation. Future studies could conduct longitudinal data analysis to examine the temporal dynamics of the relationship between political trust and public participation. Second, factors such as political efficacy play a vital role in shaping public participation. Future research could investigate additional influential factors that contribute to the explanation of the mechanism from political trust to public participation.

## Supporting information

Chinese general social survey data, CGSS. (SAV)
